# Potassium induced contraction of the internal thoracic artery in vitro is time related: the potential consequences in the analysis of the mechanism of the spasm after coronary artery bypass grafting and in the analysis of the results of in vitro studies

**DOI:** 10.1007/s00380-015-0684-y

**Published:** 2015-05-05

**Authors:** Tomasz Kleszczewski, Leszek Buzun, Anna Lisowska, Beata Modzelewska

**Affiliations:** Department of Biophysics, Medical University of Bialystok, ul. Mickiewicza 2A, 15-222 Białystok, Poland; Department of Cardiac Surgery, Medical University of Białystok, ul. M. Skłodowskiej-Curie 24A, 15-276 Białystok, Poland; Department of Cardiac Surgery, The Regional Specialist Hospital in Olsztyn Poland, ul. Żołnierska 18, 10-561 Olsztyn, Poland; Department of Cardiology, Medical University of Białystok, ul. M. Skłodowskiej-Curie 24A, 15-276 Białystok, Poland

**Keywords:** Bypass, Contractility, Depolarization, Potassium, Vasospasm

## Abstract

The aim of the present study was to examine how, under in vitro conditions, the human left internal thoracic artery (LITA) reacts to contractile agonist:potassium chloride (KCL) as a function of time, as well as to examine whether a change in the LITA reactivity can correlate with the occurrence of the refractory vascular spasm (RVS). Distal segments of LITA obtained from 33 patients aged 38–73, at the time of routine coronary artery surgical revascularization (CABG). Contractile response to 80 mmol K^+^ was recorded under isometric conditions. In 16 (48,5 %) LITA segments, contractile reaction to K^+^ after experiments ranging 1–10 h were registered. No contractile response even after 10 h of incubation was observed in 17 (51.5 %) LITA segments. Between 120 and 300 min after the beginning of the experiment, the reaction was maximum and amounted up to 300 % control reaction, then decreased. First, with respect to in vitro research isolated by LITA rings, while analyzing the results of the research, one should take into consideration the possibility that during the research, the functional state of the tissues changes and in particular its sensitivity to depolarization of the cell membrane. Second, the change in the sensitivity to depolarization of the cell membrane of the smooth muscles’ LITA might be the potential mechanism causing the occurrence of the postoperative spasm after the CABG treatment.

## Introduction

Isolated blood vessel pharmacology allows the exploration of mechanisms of drug action, and establishment of concentration–response relationships for analysis of potency and range more readily than is possible in in vivo experiments. It cannot identify the cause of in vivo vasoconstriction; however, isolated tissue assays can determine what factors have the potential to contract the vessel in vivo. The left internal thoracic artery (LITA) is a dynamic conduit used for myocardial revascularization [1–4] in which potential exists as well for spasm as for vasodilatation. There are many vasoactive agents used in pharmacologic and surgical treatment of coronary artery disease which may influence the reactivity of LITA. Some of them are described as having long-lasting effects [5–7].

Studies of LITA reactivity reported in the literature are based on in vitro measurements after 30–90 min incubation [6, 8–12]. There is a lack of information about the possible influence of preoperative pharmacology, especially long-acting vasodilators, on the incubation time needed to obtain vasoreactivity measurements. Our long-term experience in working on the LITA reactivity under in vitro conditions indicates that there are difficulties in obtaining a contractile response using different factors causing contractions even during prolonged experiments. Likely, most experimenters will exclude vessels that do not react because they take for granted that they were damaged during harvesting or during the experimental preparation. But is this assumption always correct? Our doubts emerged after preliminary trials in which the contraction induced by potassium and norepinephrine occurred even after several hours of incubation.

On the other hand, coronary artery spasm is a known complication of coronary artery bypass grafting (CABG) and can be associated with circulatory collapse and death. Refractory vascular spasm (RVS) has been reported to occur between 0.8 and 1.3 % of CABG procedures [13, 14], although transient coronary or graft spasm has been shown to affect up to 11 % of operated patients [15]. Analysis of data from two multicenter prospective studies in Italy showed, that thirty-day postoperative mortality rate among octogenarians was at 6.5 % (147/2246 patients), which was significantly higher than among patients aged under 80 years [16]. However, the incidence and mortality rates may be underestimated because mainly the surviving cases are reported and exact diagnosis can only be achieved with coronary angiography. In the cases described in the literature, confirmed by the angiographic research, the RVS occurred between 2 and 8 h after the surgery [17–21]. Thus, it seems that the greatest risk of postoperative coronary artery spasm is very early after CABG surgery and that this risk declines with time.

We have decided to verify how the LITA reactivity changes as a function of time during long-hour incubation to see what effect might it have on the results of in vitro research lead using standard procedures and whether a change in LITA reactivity might correlate with the occurrence of RVS in patients subjected to CABG.

## Materials and methods

Distal segments of LITA obtained from 33 non-diabetic patients aged 38–73, at the time of routine coronary artery surgical revascularization. This study was reviewed and approved by the local committee of the ethics on human research (the approval reference number–R-I-003/42/2002). Because the internal thoracic artery obtained during coronary artery bypass operation was classified as a surgical specimen, its use was exempted by the committee from required patient consent. The study was performed conform the declaration of Helsinki.

Patients were preoperatively long-term treated with:

1. Beta-blockers—administered just prior to surgery
  Selective (bisoprolol—half-life 17 ± 5 h),
  Non-selective (metoprolol—half-life 3.5 h);
2. ACE-inhibitor—discontinued at least 12 h prior to surgery—ramipril—The maximum effect after the administration of a single oral dose occurs normally within 3–6 h;
3. Calcium channel blocker—discontinued at least 12 h prior to surgery—amlodipine—half-life 35–50 h;
4. Nitrates—administered just prior to surgery—half-life 4–5 h.
5. Statins—discontinued at least 24 h prior to surgery—simvastatin, atorvastatin

After removal, the tissue was treated with papaverine for a period of 5–30 min.

Any obtained segments were collected and put into container with oxygenated physiologic solution maintained at 4° C (NaCl-121, KCl-4.7, NaHCO_3_-24.7, MgSO_4_-12.2, CaCl2-2.5, KH_2_PO_4_-1.2, glucose-5.8, (mmol/L)), pH 7.4, and then transferred to a laboratory immediately.

Under a dissecting microscope, arterial rings (length 2–3 mm) were prepared. The rings were mounted in an organ bath containing the physiological salt solution of pH 7,4 and at temperature 37 °C, and bubbled with carbogen (95 % O_2_ and 5 % CO_2_). The details of the technique were published previously [8, 11, 22, 23]. The experiment was started within 1–3 h of tissue recovery. At the beginning of the experiments, the rings were stretched to an initial tension of about 15 mN and allowed to relax and equilibrate for about 1 h in the bathing medium then the vascular preparations were challenged once with norepinephrine (1 µM). If there was a reaction to norepinephrine, when the effect was levelling off, the presence of functional endothelial cells was tested by adding 1 µM acetylcholine. Then, the tissues were rinsed and after 1 h, K^+^ was administered (80 mmol). In case of tissues in which there was a reaction to norepinephrine, the administration of potassium was repeated 6 times in 1-h intervals. In case the reaction to norepinephrine did not occur, the administration of potassium was repeated 10 times in 1-h intervals. Contractile response of the arterial rings was recorded under isometric conditions over 10 min. Quantification of the response was done by calculation of the area under the curve of contractions.

### Statistical analysis

The data were reported as mean ± SEM. Data were evaluated for statistical significance using two-way ANOVA for repeated measures. A probability of less than 0.05 (*p* < 0.05) was considered statistically significant.

## Results

**Fig. 1:**
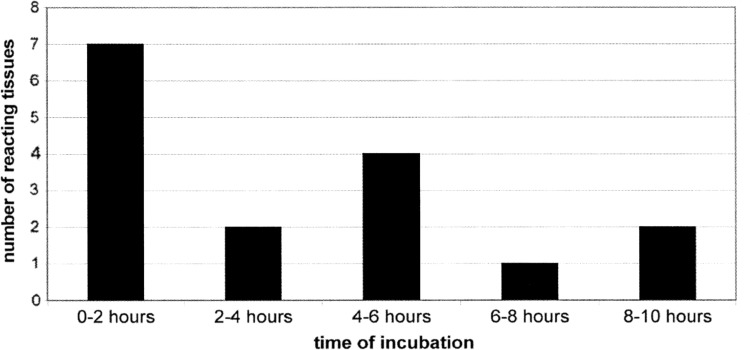
The number of reacting tissues in relation to the period of incubation

In 16 (48.5 %) LITA segments from 33 in the study group, contractile reaction to K^+^ after experiments ranging 1–10 h was registered. No contractile response even after 10 h of incubation was observed in 17 (51.5 %) LITA segments. Among 16 reacting tissues, only in 7 cases was there a reaction to norepinephrine and then to K^+^. In all those 7 cases, the reaction to acetylcholine indicated the correct endothelium function. In other 9 cases, the reaction to potassium occurred at different times from the beginning of the experiment. Figure 1 shows the number of tissues in which there was a reaction to potassium in function of the period of incubation.

Unlike the data commonly presented in the literature [8, 9, 11, 24–26], we noticed that contractile reaction of investigated LITA segments may occur even after prolonged incubation time. In our series, 56.25 % of reacting tissues showed the response after incubating for longer than 120 min. From the whole study group, no contractile response even after 10 h of incubation was observed in 17 (51.5 %) arteries.

In the case of 7 tissues in which there was a reaction to norepinephrine, the reaction to potassium was time-dependent, as Fig. 1 shows.

In the period between 120 and 300 min after the beginning of the experiment, the response to potassium was maximal and accounted for approximately 300 % of the control reaction, then decreased. Analysis of variance showed that the reactions after 120, 180, 240 and 300 min did not differed statistically significantly.

## Discussion

In in vitro LITA rings tests usually standard procedures are used—different contractile agents or vasodilators or their combinations are analyzed. Tissues used in this kind of experiment are always taken from patients subject to long-term pharmacotherapy; usually the patients are administered with beta-blockers, calcium channel blockers, nitrates, ACE-inhibitors and the experiment is conducted as soon as possible after the removal of the tissue. The question arises: how much does the tissue change during the in vitro experiment that takes up to a few hours and can one assume that during the whole experiment the tissues does not change its functional state? Only 21 % of the tested tissues, taken from different patients in our experiment have responded with a contraction to NE and then KCl administration when the incubation time was shorter than 120 min, and as much as 51.5 % of the tissues have not reacted with a contraction even after 10 h of incubation. In papers describing LIMA rings in vitro research, the authors usually quote the time of incubation between 30 and 90 min [6, 8–12]. We have not found in accessible literature any data describing experiments in which the LITA rings did not react with a contraction to NE and KC1 administration. So, either all tissues tested in different laboratories did react with a contraction to α-adrenoceptors stimulation and depolarization of the cell membrane (which in view of our long-term research on this tissue does seem unlikely) or only reacting tissues entered in the experiments and analysis. The abolition of the contractile response to norepinephrine within a few hours from the collection of LITA may be caused by pre-incubation of the tissue with α-adrenoceptors inhibitor papaverine. Harrison and associates demonstrated that the protective effects of papaverine are relatively short-lived, when the human radial artery is challenged with NE. However, this complete inhibition of constriction remained for 2 h after wash-out of papaverine and to 4 h after pre-treatment with papaverine, the contraction is 10^−5^ M NE was 21 ± 9 % of control vessels [27]. On the other hand, Mirkami and associates showed that papaverine completely relaxed NE-induced contraction of the ITA [28]. Time elapsed in our experiments since the removal of the tissue to the first administration of the NE contained within 2–5 h. So it seems that the lack of response to NE, LITA for our experiments (79 %) can be explained by the inhibitory effect of papaverine. The contractile response to KCl require in intracellular calcium which is produced in response to membrane depolarization and extracellular Ca^2+^ influx through voltage-operated Ca^2+^ channels (VOC) triggers Ca^2+^—induced Ca^2+^ release from intracellular stores [29–31]. The lack of reaction to KC1 administration can thus indicate that this way of tissue stimulation is inactive and voltage-operated Ca^2+^ channels (VOC) may be blocked. With the passage of time of the experiment, the tissues start reacting to cell membrane depolarization with a contraction (Fig. 1). Such a situation, in our opinion, is caused, above all, by preoperative pharmacotherapy and, in particular, by long-acting blockers of the voltage-operated Ca^2+^ channels (VOC)—amlodipine administration. In their work, Bai and associates have shown that [32] the pretreatment of isolated LITA with plasma concentrations of amlodipine (−6.6 log M) significantly depressed subsequent contraction to KCl. During the in vitro experiment, when the tissue is repeatedly rinsed, a part of the channel does get unblocked and the tissue obtains the ability to contract. Another factor that may affect the functioning of the cell membrane of smooth muscle cells and VOCs may be the statins administered to the patients in the preoperative period. There are many reports confirming synergistic effects of statins and calcium channel blockers [33–36]. At the same time, epidemiological studies have shown, that statin in combination with β-blocker therapy reduces postoperative stroke after CABG [37], statins reduce short- and long-term mortality associated with postoperative atrial fibrillation after CABG [38], statin therapy was associated with reduced all-cause mortality among patients with chronic kidney disease (CKD) and coronary artery disease (CAD) after percutaneous coronary intervention (PCI) [38].

**Fig. 2:**
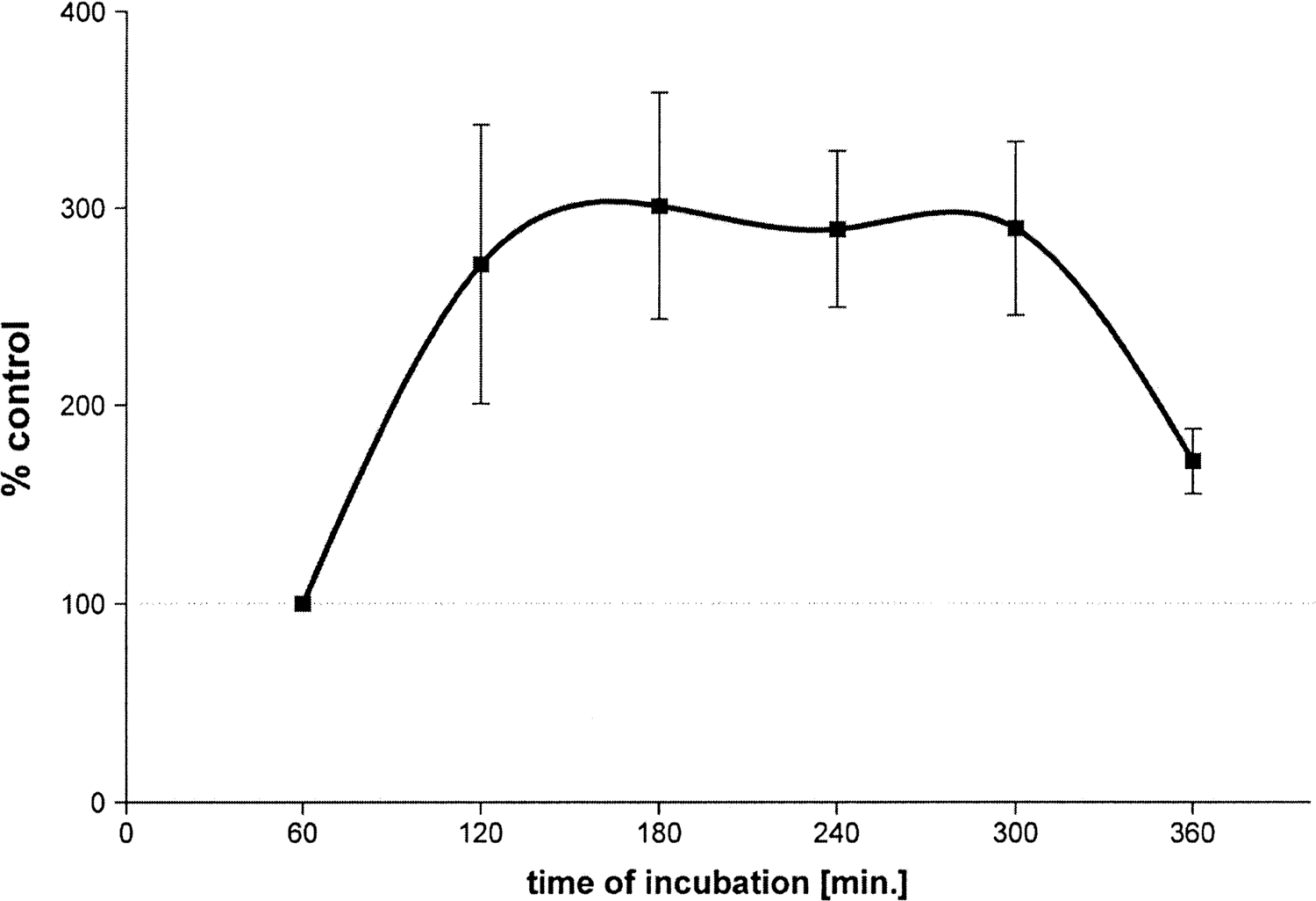
Reaction dependence on K^+^ (80 mmol) of 7 LITA rings. The control was the area under the curve for the first contraction of potassium for each tissue, respectively. *Points* represent mean values from *n* = 7 individual experiments; vertical lines represent ± s.e. mean

For the tissues, which did not react to KC1 administration in an incubation time shorter than 120 min, we have concluded that the reaction is time-dependent and at its maximum between 120 and 300 min after the beginning of the experiment (Fig. 2). Such a conclusion is suggested by the increase in calcium level inside the muscle cells during the experiment. It may be caused by the increased inflow of calcium from the intercellular space through the voltage-operated Ca^2+^ channels (VOC) as well as by the release of a greater amount of calcium from intracellular stores. In our opinion, this effect may have an influence on the results of the in vitro experiments in which the influence of various contractile agents or vasodilators, or their combination in LITA rings is analyzed and thus, such an effect should be included during the analysis of the obtained results of such research.

The research on isolated tissues may not include all the factors influencing the contractile LITA action in vivo, and in particular, the factors that cause the spasm. In this paper, we concentrated solely on the contraction caused by the depolarization of the cell membrane of the muscle cell.

The spasm cases described in the literature do occur—during the surgery at the operating room, during the transport of the patient to the recovery room or within a short period of time (2–8 h) after the CABG surgery [17–21, 39].

The time of the CABG surgery is naturally diversified and dependent on many factors. From the removal of LITA until chest closure about 60–180 min elapse. In our experiments, the time needed to move the tissue from the operation room to the laboratory, the tissue preparation, the placement of LITA rings in the incubation vessels and obtaining a stable basic voltage, is comparable with the time of a standard CABG surgery. We have not found in the accessible literature any detailed analysis of the time of the respective stages of the in vitro procedure in such research, however, on can assume, that it is similar to other laboratories. Thus, if the first administration of the vessel contractile agent may be considered as the launch of the experiment, the time is similar to the termination of the CABG surgery.

Seven out of 33 LITA rings have reacted with a contraction to KC1 administration at the beginning of the experiment, thus in a time nearing the termination of the CABG surgery. In successive 2 h time intervals of the incubation, the number of tissues in which there was a contractile reaction to KC1 administration was, respectively: 2, 4, 1 and 2. Of course this cannot be the basis for the conclusion that there is a correlation between the reaction time of the isolated LITA rings caused by the KC1 and the possibility of spasm or other cardiovascular incidents occurrence among patients after the CABG surgery. Nevertheless, in our opinion, this observation indicates that during in vitro studies of the ITA rings, the duration of each phase of the experiment, calculated since harvesting of the tissue is an important parameter that should be taken into account in the analysis of the results. This in-depth analysis would help to translate the received effects in a clinical scenario.

## Conclusions

In summation, the results of our research can be examined in two ways. First, when analyzing in vitro research of the isolated LITA rings, one should perhaps take into consideration the fact that during the experiment, the functional state of the tissue changes, in particular—its sensitivity to cell membrane depolarization. Secondly, the change in sensitivity to cell membrane depolarization of the smooth muscle LITA cells may be the mechanism influencing the post-operative spasm after the CABG surgery.
